# LAMPhimerus: A novel LAMP assay for detecting *Amphimerus* sp. DNA in human stool samples

**DOI:** 10.1371/journal.pntd.0005672

**Published:** 2017-06-19

**Authors:** William Cevallos, Pedro Fernández-Soto, Manuel Calvopiña, Cristina Fontecha-Cuenca, Hiromu Sugiyama, Megumi Sato, Julio López Abán, Belén Vicente, Antonio Muro

**Affiliations:** 1 Centro de Biomedicina, Carrera de Medicina, Universidad Central del Ecuador, Quito, Ecuador; 2 Infectious and Tropical Diseases Research Group (e-INTRO), Biomedical Research Institute of Salamanca-Research Centre for Tropical Diseases at the University of Salamanca (IBSAL-CIETUS), Faculty of Pharmacy, University of Salamanca, Salamanca, Spain; 3 Carrera de Medicina, Universidad De Las Américas (UDLA), Quito, Ecuador; 4 Department of Parasitology, National Institute of Infectious Diseases, Tokyo, Japan; 5 Graduate School of Health Sciences, Niigata University, Niigata, Japan; James Cook University, AUSTRALIA

## Abstract

**Background:**

Amphimeriasis is a fish-borne disease caused by the liver fluke *Amphimerus* spp. that has recently been reported as endemic in the tropical Pacific side of Ecuador with a high prevalence in humans and domestic animals. The diagnosis is based on the stool examination to identify parasite eggs, but it lacks sensitivity. Additionally, the morphology of the eggs may be confounded with other liver and intestinal flukes. No immunological or molecular methods have been developed to date. New diagnostic techniques for specific and sensitive detection of *Amphimerus* spp. DNA in clinical samples are needed.

**Methodology/Principal findings:**

A LAMP targeting a sequence of the *Amphimerus* sp. internal transcribed spacer 2 region was designed. *Amphimerus* sp. DNA was obtained from adult worms recovered from animals and used to optimize the molecular assays. Conventional PCR was performed using outer primers F3-B3 to verify the proper amplification of the *Amphimerus* sp. DNA target sequence. LAMP was optimized using different reaction mixtures and temperatures, and it was finally set up as LAMPhimerus. The specificity and sensitivity of both PCR and LAMP were evaluated. The detection limit was 1 pg of genomic DNA. Field testing was done using 44 human stool samples collected from localities where fluke is endemic. Twenty-five samples were microscopy positive for *Amphimerus* sp. eggs detection. In molecular testing, PCR F3-B3 was ineffective when DNA from fecal samples was used. When testing all human stool samples included in our study, the diagnostic parameters for the sensitivity and specificity were calculated for our LAMPhimerus assay, which were 76.67% and 80.77%, respectively.

**Conclusions/Significance:**

We have developed and evaluated, for the first time, a specific and sensitive LAMP assay for detecting *Amphimerus* sp. in human stool samples. The procedure has been named LAMPhimerus method and has the potential to be adapted for field diagnosis and disease surveillance in amphimeriasis-endemic areas. Future large-scale studies will assess the applicability of this novel LAMP assay.

## Author summary

Amphimeriasis, a fish-borne zoonotic disease caused by the liver fluke *Amphimerus* spp., is a highly prevalent parasitic infection affecting an indigenous Amerindian group, the Chachi, living in rural and remote tropical areas along the Río Cayapas and its tributaries in the north-western coastal rainforest of Ecuador. Very little is known about the clinical course and treatment of this disease, and the only method for diagnosing it is the parasitological microscopic detection of eggs from *Amphimerus* spp. in patients' stool samples. This method lacks sensitivity, and the morphology of the eggs may be confounded with other liver and intestinal flukes. New diagnostic tools that can improve the sensitivity and specificity for diagnosing *Amphimerus* spp. infection would be desirable. At present, LAMP technology shows all the characteristics required of a real-time assay with simple operation for potential use in the clinical diagnosis of infectious diseases, particularly in the field conditions in developing countries for most neglected tropical diseases. In this study, we developed and successfully evaluated a LAMP assay for detecting *Amphimerus* sp. in human stool samples. After further validation, our LAMP assay (LAMPhimerus) could be readily adapted for effective field diagnosis and disease surveillance in amphimeriasis-endemic areas.

## Introduction

*Amphimerus* spp. are digenean parasitic flatworms in the bile ducts of birds, reptiles and mammals, and they are closely related to the genera *Clonorchis* and *Opisthorchis* within the Opisthorchiidae family [1, 2]. As for other members of the Opisthorchiidae family, the life cycle of *Amphimerus* spp. is highly complex, involving both freshwater snails and fish as intermediate hosts and vertebrates, including humans, as definitive hosts [3]. Humans or fish-eating animals are infected with *Amphimerus* spp. through the ingestion of raw or undercooked freshwater fish containing metacercariae [3]. Recently, *Amphimerus* sp. has been reported, for the first time, as endemic in rural communities in the tropical Pacific side of Ecuador with a high prevalence in humans and domestic cats and dogs, causing amphimeriasis [3, 4]. Several foodborne trematodiases around the world are now considered by the World Health Organization as neglected tropical diseases (NTDs) [5] with high prevalence, especially in East Asia [6], and they have serious consequences, such as cholangiocarcinoma [7,8]. Amphimeriasis has been reported as a new emerging foodborne zoonotic disease [3].

*Amphimerus* spp. adult stages are located in the bile ducts of the definitive host, and the eggs are shed in the feces [3]. Diagnosis of human and animal infection can be performed with the wet mount technique for examining feces, allowing for microscopic visualization of parasite eggs; the formalin-ether concentration method has been shown to increase the sensitivity ten-fold [3]. Detection of the eggs in bile or duodenal fluid can also be performed. However, microscopic examination is cumbersome and time consuming, and it could have a low sensitivity in cases of light infections. In addition, the morphological similarity of the *Amphimerus* spp. eggs to those of closely related species belonging to genera *Clonorchis* and *Opisthorchis* as well as to minute intestinal flukes, makes diagnosis difficult. It would be necessary to use scanning electron microscopy to accurately observe the differences between the coatings of the different species [3]. Therefore, the development of a new method that can improve the sensitivity and specificity for diagnosing *Amphimerus* spp. infection is urgently required.

To overcome these limitations, the use of molecular approaches has become a powerful tool for the diagnosis, identification and differentiation of closely related species. In recent years, several polymerase chain reaction (PCR)-based molecular diagnostic methods have been developed for detecting many parasitic trematodes, including those species that are closely related to *Amphimerus* spp., such as *C*. *sinensis* [9–14] and *O*. *viverrini* [15–18]. Although these studies have demonstrated that PCR-based methods are very sensitive and specific, they are not still widely used in low-income countries because well-trained personnel and expensive equipment are needed, making them unviable for routine application in field conditions in endemic areas that are generally undeveloped and have a high disease prevalence. Loop-mediated isothermal amplification (LAMP) could be a good alternative amplification technology [19] because it has several salient advantages over most PCR-based methods [20, 21]. At present, LAMP technology has all the characteristics required of a real-time assay along with simple operation for potential use in the clinical diagnosis of infectious diseases, particularly under the field conditions in developing countries [22, 23]. Additionally, several LAMP assays have already been successfully described for detecting trematode parasites, including a number of species causing foodborne trematodiases, such as *Fasciola* spp. [24], *Clonorchis sinensis* [25, 26], *Opisthorchis viverrini* [27–29] and *Paragonimus westermani* [30].

With the aim of developing new, applicable and cost-effective molecular tools for the diagnosis of amphimeriasis, we have developed and evaluated, for the first time, a LAMP assay for the specific detection of *Amphimerus* sp. liver fluke in human stool samples.

## Methods

### Ethics statement

The study protocol was approved by the Ethics Committee of Universidad Central del Ecuador (License number: LEC IORG 0001932, FWA 2482, IRB 2483. COBI-AMPHI-0064-11) and the Ethics Committee of the University of Salamanca (protocol approval number 48531). Participants were given detailed explanations about the aims, procedures and possible benefits of the study. Written informed consent was obtained from all subjects prior to the collection of biological samples for parasitological and molecular evaluation. Parents or guardians of children who participated in the study provided written informed consent on the child's behalf. All samples were coded and treated anonymously.

### Study area and population

The study was conducted during February 2016 in two indigenous Chachi villages alongside the Cayapas River in the Esmeraldas province, located in the northwest coastal rainforest of Ecuador [4]. The indigenous Chachi, living together with the Afro-ecuadorian and mestizo populations, belong to the predominant autochthonous group in this area, representing 13% of the inhabitants in this region. These communities are the same as those studied previously and have a high prevalence of infection (15.5% to 34.1%) with *Amphimerus* sp. Prevalences are also high in local cats and dogs [3, 4]. They live in remote villages where the only way to reach them is by boat along the river. Sanitation facilities are lacking, and the members are hunters who habitually eat undercooked freshwater fish (mainly smoked fish) caught in the neighboring rivers [4]. More details on the region can be accessed elsewhere [31, 32].

### Human stool samples and parasitological tests

Human stool samples were obtained from indigenous Chachi communities during February 2016. Each participant who enrolled in the study was given a copro-parasitological flask for stool collection. Samples were collected within a few hours of stool passing. After collection, samples were transported to the Parasitology Laboratory (Centro de Biomedicina, Universidad Central del Ecuador, Quito, Ecuador) for parasitological screening under light microscopy by direct examination, simple sedimentation, formalin-ether concentration and Kato-Katz techniques. All samples were examined by two qualified laboratory technicians according to the basic laboratory methods in medical parasitology recommended by the World Health Organization (WHO) [33]. After parasitological screening, a total of 44 stool samples were selected, including 25 (56.81%) that were positive for *Amphimerus* sp. eggs-by one or more parasitological methods-and 19 (43.18%) negative samples. Afterwards, the 44 stool samples that were well-preserved in 80% ethanol were sent to the Research Center for Tropical Diseases (CIETUS) at the University of Salamanca, Spain, for further DNA extraction and molecular analysis as described below.

### DNA extraction for molecular analyses

#### DNA from human fecal samples

Approximately 250–300 mg from each of 44 stool samples preserved in 80% ethanol solution was used for DNA extraction. First, excess ethanol was removed from each vial; subsequently, DNA extraction was performed using the Mini Stool DNA Extraction kit (Macharey-Nagel) according to the manufacturers’ instructions. Purified DNA samples were stored at -20°C until use.

#### DNA from parasites

*Amphimerus* sp. genomic DNA was extracted from frozen adult worms that were previously obtained from the livers of naturally infected cats and dogs of Chachi communities, as described elsewhere [4], using a G-spin Total DNA Extraction Kit (Intron Biotechnology) according to the manufacturers’ instructions. DNA was measured using a Nanodrop ND-100 spectrophotometer (Nanodrop Technologies) and then diluted with ultrapure distilled water to final concentrations of 5 ng/μL and 0.5 ng/μL. Serial 10-fold dilutions from adult *Amphimerus* sp. DNA were prepared with ultrapure water, ranging from 1x10^-1^ to 1x10^-9^, and stored at -20°C until use. DNA thus prepared was used as a positive control in all PCR and LAMP reactions as well as for assessing the sensitivity of both molecular assays.

To determine the specificity of PCR and LAMP assays to amplify only *Amphimerus* sp. DNA, a total of 16 DNA samples from several helminths, including trematodes (*Clonorchis sinensis*, *Opisthorchis viverrini*, *Fasciola hepatica*, *Dicrocoelium dendriticum*, *Schistosoma mansoni*, *S*. *haematobium*, *S*. *japonicum*, and *S*. *intercalatum*), cestodes (*Echinococcus granulosus* and *Taenia truncata*), nematodes (*Onchocerca volvulus*, *Strongyloides venezuelensis*, and *Trichinella spiralis*) and protozoa (*Entamoeba histolytica*, *Cryptosporidium parvum*, and *Giardia duodenalis*) were used. The concentration of all DNA samples was measured by the same method as described for *Amphimerus* sp. DNA, which was then diluted with ultrapure water to a final concentration of 0.5 ng/μL and kept at -20°C until use in molecular assays.

### Designing LAMP primers

**Fig 1:**
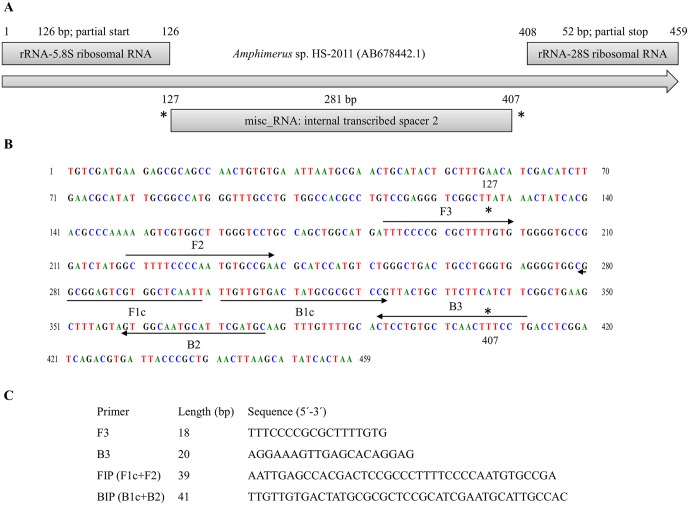
Design of LAMP primers for detecting DNA of *Amphimerus* sp. (A) Schematic representation of the 459 bp selected sequence of *Amphimerus* sp. HS-2011 isolated from human host (AB678442.1). (B) Location of the LAMP primers within the selected sequence. Arrows indicate the direction of extension. (C) Sequences of LAMP primers. F3, forward outer primer; B3, reverse outer primer; FIP, forward inner primer (F1c and F2 sequences); and BIP, reverse inner primer (B1c and B2 sequences).

An 459 base pair (bp) sequence, corresponding to a linear genomic DNA partial sequence in the ITS2 region of *Amphimerus* sp. HS-2011 isolated from human host, was selected and retrieved from GenBank (Accession No. AB678442.1) [4] for the design of the specific primers. The 459 bp sequence was tested using BLASTN analysis [34] for similarity in the available online genome databases. A set of LAMP primers complementary to the nucleotide sequence was designed using the online Primer Explorer V4 software (https://primerexplorer.jp/elamp4.0.0/; Eiken Chemical Co., Ltd., Tokyo, Japan) according to criteria described by Notomi et al [19]. A final complete set of four primers-including a forward outer primer (F3), a reverse outer primer (B3), a forward inner primer (FIP) and a backward inner primer (BIP)-was selected based on the criteria described in “A guide to LAMP primer designing” (http://primerexplorer.jp/e/v4_manual/index.html) of LAMP primers; the locations and target sequence are shown in Fig 1. All the primers were of HPLC grade (Thermo Fisher Scientific Inc., Madrid, Spain). The lyophilized primers were resuspended in ultrapure water to a final concentration of 100 pmol/μL and stored at -20°C until use.

### PCR using outer primers F3 and B3

The outer LAMP primer pair (F3 and B3; Fig 1) was initially tested for *Amphimerus* sp. specificity by a PCR to verify whether the correct target was amplified. PCR was conducted in 25 μL of a reaction mixture containing 2.5 μL of 10x buffer, 1.5 μL of 25 mmol/L MgCl_2_, 2.5 μL of 2.5 mmol/L dNTPs, 0.5 μL of 100 pmol/L F3 and B3, 2 U *Taq*-polymerase and 2 μL (10 ng) of DNA template. Initial denaturation was conducted at 94°C for 1 min, which was followed by a touchdown program for 15 cycles with successive annealing temperature decrements of 1.0°C every 2 cycles. For these 2 cycles, the reaction was denatured at 94°C for 20 s followed by annealing at 64°C-58°C for 20 s and polymerization at 72°C for 30 s. The subsequent 15 cycles of amplification were similar, except that the annealing temperature was 57°C. The final extension was performed at 72°C for 10 min. All PCR reactions were performed in a Mastercycler Gradient-96well (Eppendorf).

The specificity of PCR F3-B3 was tested using heterogeneous DNA samples from other parasites included in the study. The sensitivity was also assayed to establish the detection limit of *Amphimerus* sp. DNA with 10-fold serial dilutions prepared as mentioned above. All PCR assays were performed with 2 μL of the DNA template (5 ng/μL) in each case. Negative controls (ultrapure water) and positive controls (genomic DNA from *Amphimerus* sp.) were always included. The PCR products (3–5 μL/each) were subjected to 1.5–2% agarose gel electrophoresis stained with ethidium bromide and visualized under UV light.

### Establishing the LAMP assay

We evaluated the LAMP primer set designed by using different reaction mixtures to compare results in *Amphimerus* sp. DNA amplification. LAMP reactions mixtures (25 μL) contained 40 pmol each of FIP and BIP primers, 5 pmol each of F3 and B3 primers, 1.4 mM each of dNTP (Intron), 1x Isothermal Amplification Buffer-20 mM Tris-HCl (pH 8.8), 50 mM KCl, 10 mM (NH_4_)_2_SO_4_, 2 mM MgSO_4_, 0.1% Tween20 (New England Biolabs, UK)-betaine (0.8, 1, 1.2, 1.4 or 1.6 M) (Sigma, USA), supplementary MgSO_4_ (2, 4, 6 or 8 mM) (New England Biolabs, UK) and 8 U of *Bst* polymerase 2.0 WarmStart (New England Biolabs, UK) with 2 μL (1 ng) of template DNA.

LAMP reactions were performed in 0.5-mL micro-centrifuge tubes that were incubated in a simple heating block at a range of temperatures (61, 63 and 65°C) for 60 min to optimize the reaction conditions and then heated at 80°C for 5–10 min to terminate the reaction. The optimal temperature was determined and used in the following tests. Because of the high sensitivity of the LAMP reaction, DNA contaminations were prevented using sterile tools at all times, performing each step of the analysis in separate work areas and minimizing manipulation of the reaction tubes. Template DNA was replaced by ultrapure water as a negative control in each LAMP reaction.

The specificity of the LAMP assay to amplify only *Amphimerus* sp. DNA was tested against 16 DNA samples obtained from other parasites used as heterogeneous controls, as mentioned above. To determine the lower detection limit of the LAMP assay, genomic DNA from *Amphimerus* sp., 10-fold serial diluted as mentioned above, was subjected to amplification compared with the PCR F3-B3.

### Detection of LAMP products

The LAMP amplification results could be visually inspected by adding 2 μL of 1:10 diluted 10,000X concentration fluorescent dye SYBR Green I (Invitrogen) to the reaction tubes. Green fluorescence was clearly observed in the successful LAMP reaction, while it remained original orange in the negative reaction. In addition, the LAMP products (3–5 μL) were monitored using 1.5–2% agarose gel electrophoresis stained with ethidium bromide, visualized under UV light and then photographed using an ultraviolet Gel documentation system (UVItec, UK).

### Statistical analysis

To estimate the accuracy of the LAMP assay method as a diagnostic test, the percentages of the sensitivity, specificity, positive predictive value (PPV) and negative predictive value (NPV) were calculated using the MedCalc statistical program version 16.8.4 (MedCalc Software, Ostende, Belgium) according to the software instruction manual (www.medcalc.org).

## Results

### PCR using outer primers F3-B3: Sensitivity and specificity

**Fig 2:**
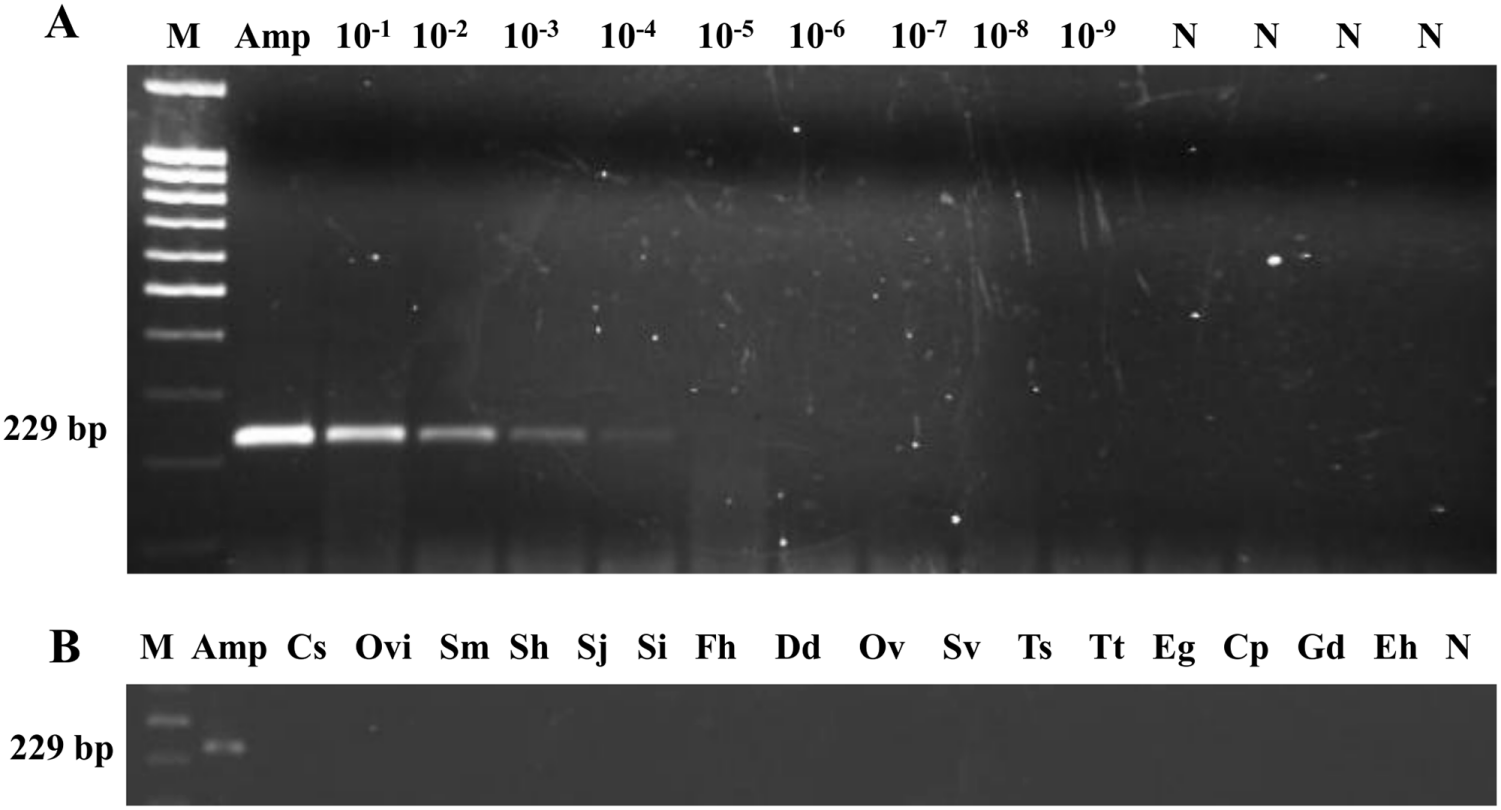
PCR verification, detection limit and specificity using outer primers F3 and B3 for DNA amplification of *Amphimerus* sp. (A) Detection limit of PCR F3-B3. Lane Amp, DNA of *Amphimerus* sp. (10 ng); lanes 10^−1^–10^−9^, 10-fold serial dilutions of *Amphimerus* sp. DNA. (B) Specificity PCR F3-B3. Lanes Amp, Cs, Ovi, Sm, Sh, Sj, Si, Fh, Dd, Ov, Sv, Ts, Tt, Eg, Cp, Gd, Eh: DNA samples of *Amphimerus* sp., *Clonorchis sinensis*, *Opisthorchis viverrini*, *Schistosoma mansoni*, *S*. *haematobium*, *S*. *japonicum*, *S*. *intercalatum*, *Fasciola hepatica*, *Dicrocoelium dendriticum*, *Onchocerca volvulus*, *Strongyloides venezuelensis*, *Trichinella spiralis*, *Taenia truncata*, *Echinococcus granulosus*, *Cryptosporidium parvum*, *Giardia duodenalis* and *Entamoeba histolytica*, respectively. In all panels: lane M, molecular weight marker (100 bp Plus Blue DNA Ladder) and lane N, negative controls (ultrapure water, no DNA template).

The expected 229 bp PCR product was successfully obtained with outer primers F3 and B3 from *Amphimerus* sp. DNA. According to sensitivity, the minimum level of *Amphimerus* sp. DNA detectable by PCR was 0.001 ng (1 pg) (Fig 2A). Additionally, when DNA samples from other parasites included in the study were subjected to this PCR assay, no amplicons were obtained (Fig 2B).

### Examination of human stool samples by PCR F3-B3

We tested the 44 human stool samples by PCR using the outer primers F3 and B3, and very faint bands of the expected size (229 bp) were only obtained in 3 samples (nos. 31, 34 and 45) (S1 Fig).

### Establishing the LAMP assay: LAMPhimerus

**Fig 3:**
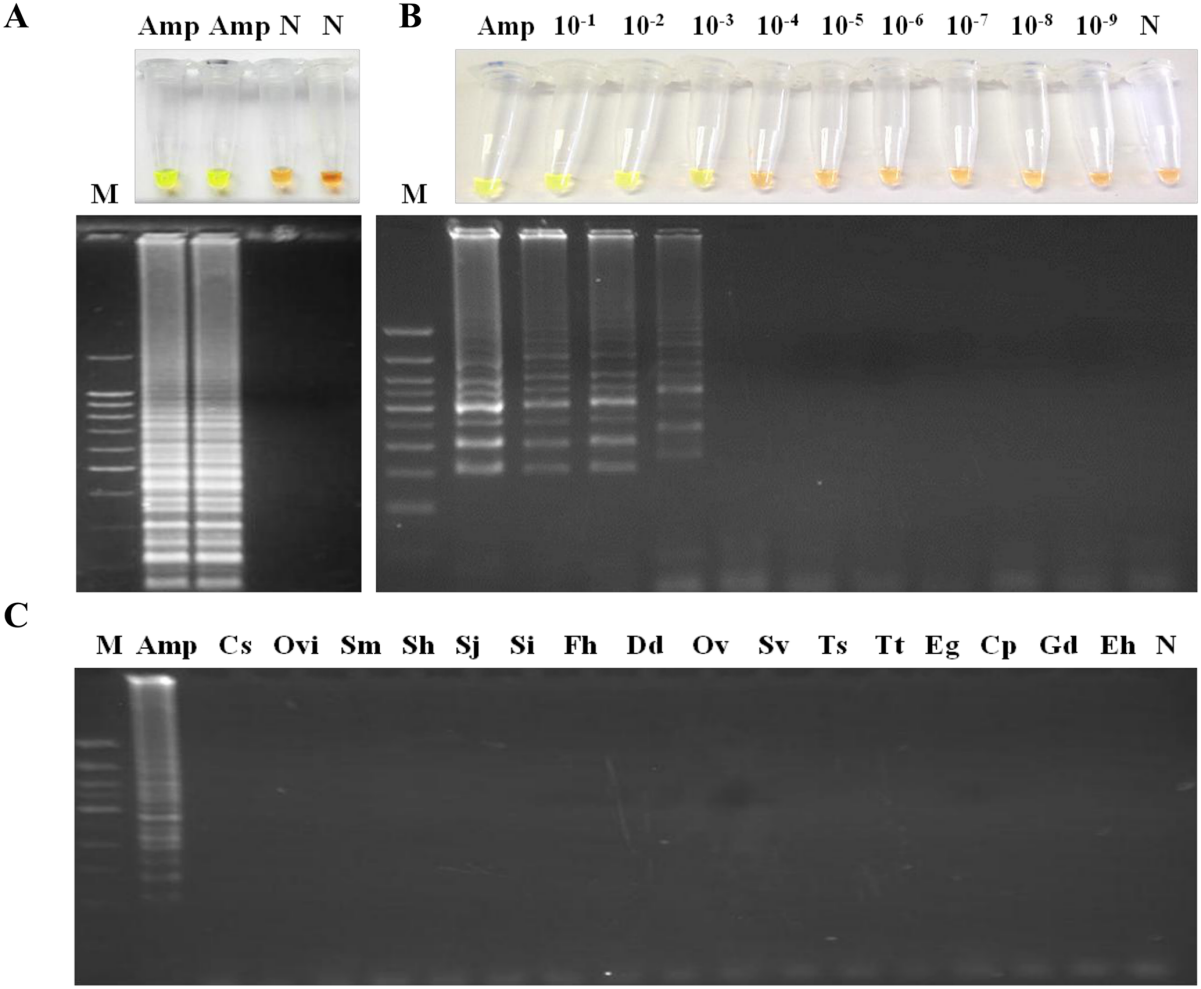
Establishing the LAMP assay. (A) LAMP amplification results obtained using the established LAMPhimerus assay with the addition of SYBR Green I (up) or visualization on agarose gel (down). Lane M, molecular weight marker (100 bp Plus Blue DNA Ladder); lane Amp, *Amphimerus* sp. DNA (1 ng); and lane N, negative control (ultrapure water and no DNA template). (B) Sensitivity assessment of LAMPhimerus using serial dilutions of *Amphimerus* sp. genomic DNA. Lane M, molecular weight marker (100 bp Plus Blue DNA Ladder); lane Amp, *Amphimerus* sp. DNA (1 ng); lanes 10^−1^–10^−9^, 10-fold serial dilutions; and lane N, negative control (ultrapure water and no DNA template). (C) Specificity of the LAMPhimerus assay. Lane M, molecular weight marker (100 bp Plus Blue DNA Ladder); lane Amp, Cs, Ovi, Sm, Sh, Sj, Si, Fh, Dd, Ov, Sv, Ts, Tt, Eg, Cp, Gd, and Eh: DNA samples of *Amphimerus* spp., *Clonorchis sinensis*, *Opisthorchis viverrini*, *Schistosoma mansoni*, *S*. *haematobium*, *S*. *japonicum*, *S*. *intercalatum*, *Fasciola hepatica*, *Dicrocoelium dendriticum*, *Onchocerca volvulus*, *Strongyloides venezuelensis*, *Trichinella spiralis*, *Taenia truncata*, *Echinococcus granulosus*, *Cryptosporidium parvum*, *Giardia duodenalis* and *Entamoeba histolytica*, respectively; and lane N, negative control (ultrapure water and no DNA template).

Subsequent to testing different reaction mixtures and temperature conditions, the best amplification results (based on the most evident color change by adding the fluorescent dye and the intensity of the multiple bands on agarose as well as reproducibility of tests) were always obtained when the LAMP master mixture contained 1 M betaine combined with supplementary 6 mM MgSO_4_ (resulting in a final concentration of 8 mM MgSO_4_ in 1x Isothermal Amplification Buffer) and was incubated at 63°C for 60 min in a heating block (Fig 3A). When we evaluated the sensitivity of the established LAMP assay, the limit of detection in *Amphimerus* sp. genomic DNA amplification was identical to that obtained when using PCR with outer primers, specifically 0.001 ng (1 pg) (Fig 3B). To determine the specificity of the LAMP assay for *Amphimerus* sp., a panel of 16 additional DNA samples from other parasites was tested for amplification. A positive result was only obtained when *Amphimerus* sp. DNA was used as template, while DNA samples from other specimens were not amplified, demonstrating its high specificity (Fig 3C).

In this way, the best reaction mixture, in addition to the specific primers designed, was established as the most fitting assay for amplification of *Amphimerus* sp. DNA and was named "LAMPhimerus" in all successive LAMP reactions.

### Application of LAMP in human stool samples: LAMPhimerus analysis

**Fig 4:**
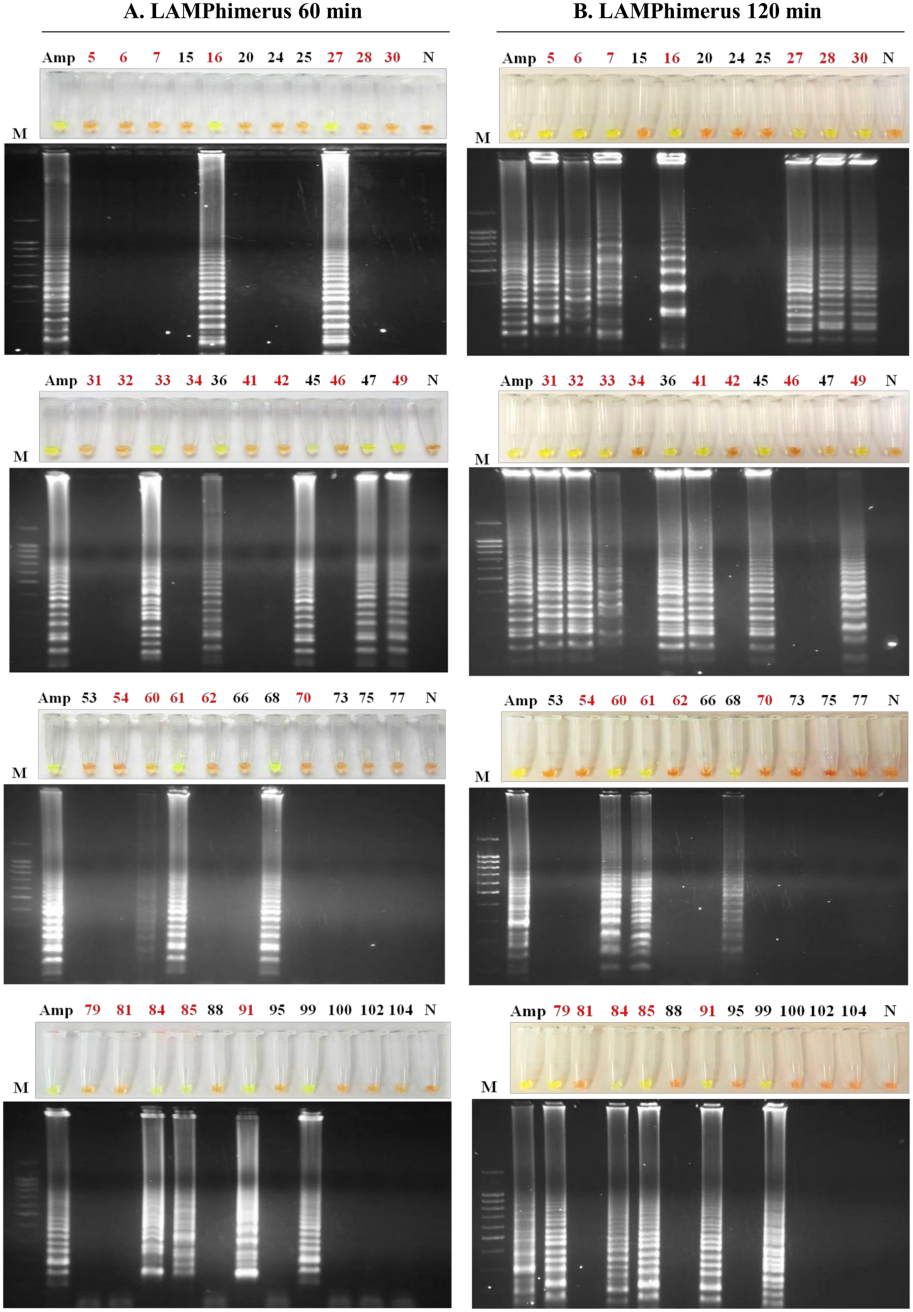
LAMPhimerus analysis of human stool samples in this study. (A) Incubation time of 60 min. (B) Incubation time of 120 min. Lane M, molecular weight marker (100 bp Plus Blue DNA Ladder); lane Amp, *Amphimerus* sp. DNA (1 ng); lane N, negative controls (ultrapure water and no DNA template); and numbers 5–104, analyzed human stool samples. The highlighted red numbers correspond to samples that were positive by one or more applied parasitological methods.

The 44 human stool samples were tested with LAMPhimerus assay using two incubation times for reaction, 60 min and 120 min (Fig 4). To prevent potential cross-contamination, amplification assays were performed in four batches of 11 samples each for easy handling. When testing stool samples using an incubation time of 60 min (Fig 4A), we obtained LAMP positive results in 14/44 (31.81%) samples, including 5 samples (nos. 36, 45, 47, 68 and 99) that were negative in all parasitological tests applied. When using an incubation time of 120 min (Fig 4B), the number of positive results was increased to 22/44 (50%), which also included 4/5 negative parasitological samples as before (nos. 36, 45, 68 and 99). In all LAMP positive reactions, green fluorescence was clearly visualized under natural light conditions. Positive controls always worked well and negative controls were never amplified. All positive results obtained when performing the assay for 60 min were supported at 120 min, except one sample (no. 47). For 120 min, in sample no. 47, a mix between green and orange was observed in the reaction tube; also a very faint smear was visible on agarose gel, indicating poor DNA amplification. Taking together the results obtained from the two incubation assays, we finally considered sample no. 47 as positive, resulting in a total of 23/44 (52.27%) positive LAMPhimerus results.

**Fig 5:**
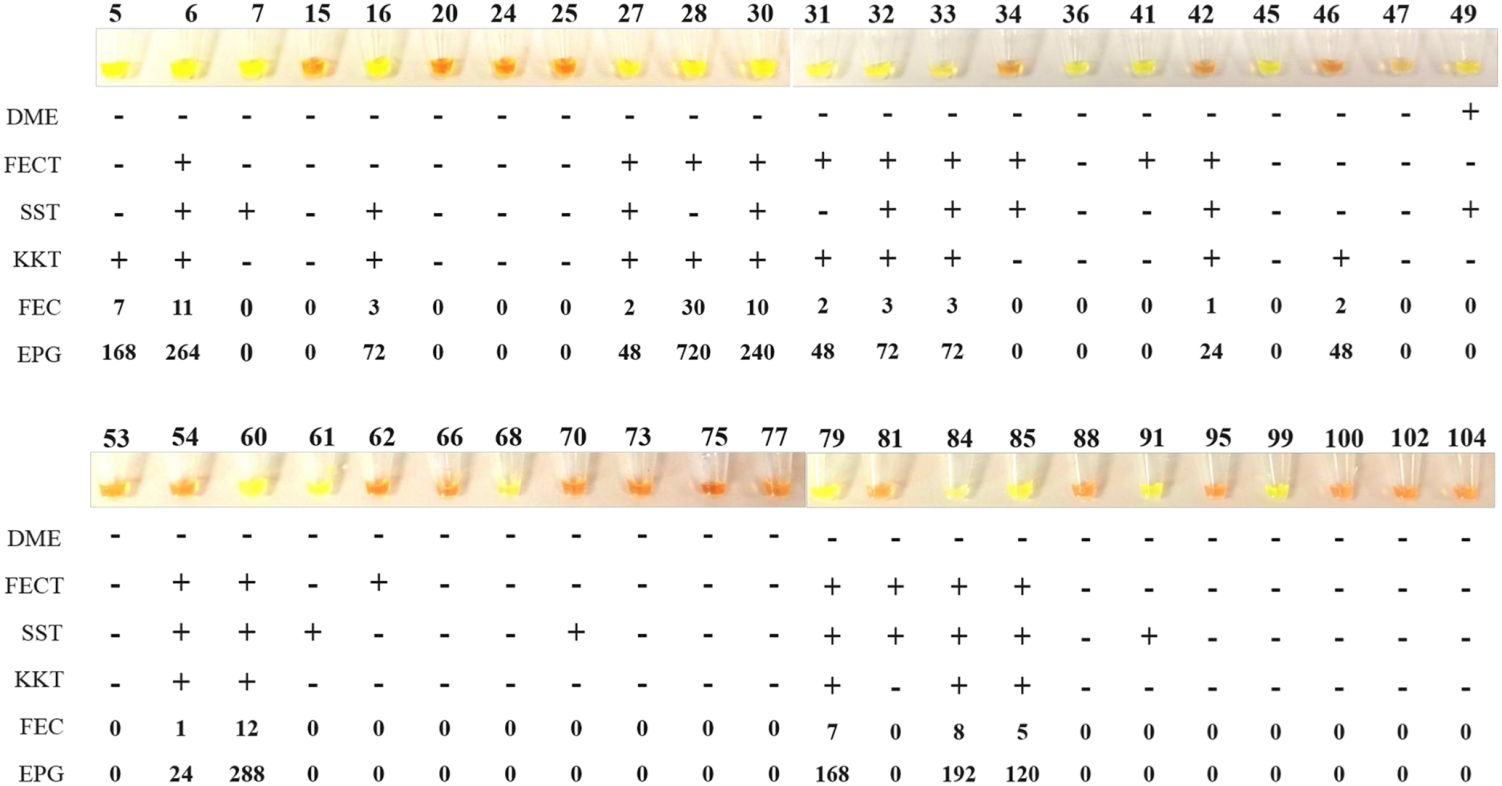
Comparison of the results obtained by the LAMPhimerus assay and classical parasitological techniques applied in this study for detecting *Amphimerus* sp. in human stool samples. DME, direct microscopy detection; FECT, formalin-ether concentration technique; SST, simple sedimentation technique; KKT, Kato-Katz technique; FEC, fecal egg count; EPG, eggs per gram of feces; -, negative for egg detection; and +, positive for egg detection. Values indicated for FEC and EPG correspond to the numbers of detected eggs and numbers 5–104 correspond to the analyzed human stool samples.

In summary, of the total of 25 parasitologically positive stool samples, we obtained 9/25 (36%) and 18/25 (72%) positive results when we applied LAMPhimerus for 60 min and 120 min, respectively. Additionally, positive results included 5/19 (26.31%) samples (nos. 36, 45, 47, 68 and 99) that were negative in all previously applied parasitological tests. Of the 11 samples (nos. 6, 27, 30, 32, 33, 42, 54, 60, 79, 84, and 85) that were simultaneously positive on three parasitological tests (including the formalin-ether concentration technique, FECT; simple sedimentation technique, SST; and Kato-Katz technique, KKT), 9 (9/11; 82%) were also positive on the LAMP assay; only the 2 samples (nos. 42 and 54) with the same very low egg count (FEC = 1; EPG = 24) were negative on the LAMP assay. Fig 5 shows a comparison of the results obtained for detecting *Amphimerus* sp. in human stool samples when using the classical parasitological techniques applied and the 120 min-LAMPhimerus assay.

Considering the results obtained, the following diagnostic parameters for the sensitivity and specificity were calculated for our LAMPhimerus assay: 76.67% sensitivity (95% CI: 52.72% -90.07%); 80.77% specificity (95% CI: 60.65% -93.45%); 82.14% positive predicted value (95% CI: 67.13% -91.20%) and 75.00% negative predicted value (95% CI: 60.43% -85.49%).

## Discussion

Human amphimeriasis, caused by the *Amphimerus* spp. liver fluke, has been recently reported as an emerging zoonotic food-borne trematodiasis [3, 4]. The conventional diagnosis of liver fluke infections in humans is based on the demonstration of eggs in different clinical samples, especially in feces. However, the morphological identification of eggs is troublesome in endemic areas where co-infection with other zoonotic trematodes usually exists. Additionally, stool examination lacks analytical sensitivity, particularly for light infections, requiring serial fecal sampling and an intensive effort in resource-poor settings [35]. To solve these limitations, many immunological and molecular diagnostic approaches have already been developed and applied to detect the presence of several human zoonotic trematode infections with varying accuracy [36, 37].

For detecting *Amphimerus* spp. infection, conventional coprological techniques are the only ones available, and no immunological or molecular methods have been developed to date. Among the possible molecular methods to be developed, LAMP tests are rapidly becoming an attractive diagnostic option for use under field conditions in laboratories with basic facilities [22, 23]. Hence, in this study, with the aim of improving the diagnostic testing for amphimeriasis, we have developed and evaluated, for the first time, a specific LAMP assay to detect *Amphimerus* sp. DNA in field samples collected from humans.

At present, nucleotide information for *Amphimerus* spp. DNA is very scarce, and only a few DNA partial sequences, corresponding to five isolates from hosts (including human, dog, cat, and two softshell turtles), are available in the Genbank database for potential LAMP primers design. The 459 bp sequence of the ribosomal DNA ITS2 region of *Amphimerus* sp. HS-2011 isolated from human hosts [4] was selected as a target of amplification. This sequence matches those later reported for isolates from a dog (dog-2012) and cat (cat-2012) residing in the same studied endemic indigenous Chachi communities for human amphimeriasis [4]. Therefore, the selection of the target region was appropriate because it seems to contain an identical sequence for all geographical isolates of *Amphimerus* spp. circulating in the same area, and the assay could be suitable for easily diagnosing both infected animals and humans in endemic areas of amphimeriasis with limited resources.

First, we established the proper operation, sensitivity and specificity of both conventional PCR (using the outer primers) and the LAMP assay (using four specific primers: LAMPhimerus) in the amplification of the *Amphimerus* sp. DNA target sequence. Both assays were shown to be highly specific for *Amphimerus* sp. because no cross-reactivity could be observed when DNA from other parasites, including those closely related such as *C*. *sinensis* and *O*. *viverrini*, were used as a template in the reactions. Identical sensitivities (1 pg of parasite genomic DNA) were obtained for both PCR and LAMPhimerus although the LAMP technique is usually 10–100 fold more sensitive than PCR [38]. However, the sensitivity obtained was the same as that previously reported for *O*. *viverrini* detection targeting the ITS1 region in rDNA (ITS1-LAMP) [27, 29] or the mitochondrial *nad1* sequence (mito-OvLAMP) [28]. A higher sensitivity (10^−5^ pg) has been reported for detecting *C*. *sinensis* targeting the cathepsin B3 gene [12]. Perhaps, in this study, a greater sensitivity could have been achieved for our LAMPhimerus assay if other DNA target sequences for designing LAMP primers had been available to analyze in databases.

When PCR was specifically tested with the 44 field-collected stool samples, only 3 very faint PCR-positive results were obtained. Varying sensitivity of PCR detection for *O*. *viverrinii* [27] and *C*. *sinensis* [14] has already been noted when analyzing human stool samples because Taq DNA polymerase inhibitors are frequent in stool specimens. Substances typically present in human feces and dietary components can also limit DNA extraction success [39]. Therefore, improvement of DNA preparation before extraction from stool samples could be a key factor for obtaining better PCR results in *Amphimerus* sp. DNA detection, as has been previously described for other similar parasites, such as *O*. *viverrini* [40, 27]. In our study, the PCR assay is not emphasized because of its very low performance and inconvenience of application in poorly equipped and often short-staffed laboratories in endemic areas.

Better results were obtained when LAMPhimerus method was applied to test human stool samples. A better performance of LAMP assays over conventional PCR methods when analyzing stool samples has been widely reported in the literature because LAMP is more tolerant to sample-derived inhibitors than PCR for diagnostic applications [41, 42]. Therefore, using the initial established reaction time of 60 min, we obtained 14/44 (31.81%) positive results, including 9/25 (36%) that tested positive by microscopy. It has been already suggested that a longer incubation reaction time in the LAMP assay improves the sensitivity and that LAMP negative samples should be incubated longer to reduce false negatives [43]. According to this, a subsequent increase to 120 min of the standard incubation time protocol for the LAMPhimerus assay allowed us to increase the number of positives results up to 23/44 (52.27%), including 18/25 (72%) microscopy-confirmed *Amphimerus* sp. infections. It should be noted that 5 stool samples with no parasite eggs (nos. 36, 45, 47, 68 and 99) were positive on LAMPhimerus testing regardless of the reaction time used for amplification. These samples could be truly *Amphimerus* sp. infections that have been microscopically undetected because of the classically low sensitivity of the parasitological diagnosis [35]. Moreover, up to 10 samples without egg counts were also LAMPhimerus positive. This result confirms a greater sensitivity of the LAMPhimerus assay over microscopic examination.

By contrast, 7 truly microscopy *Amphimerus*-positive samples (nos. 34, 42, 46, 54, 62, 70 and 81) were never amplified. For these samples, values of FEC using the Kato-Katz thick smear method were minimal (between zero and 1–2 eggs), resulting in very low EPG levels. The absence of amplification in these samples was likely not due to the ineffectiveness of LAMPhimerus method because we obtained positive results in other microscopy-positive samples with low EPG levels too. A possibility for the lack of amplification could have been the small quantity (250–300 mg) of the field-collected stool samples finally used for DNA extraction in the laboratory for the LAMP assay. Because eggs of parasites are not equally distributed among the stool specimens [44], it is possible that eggs could have been easily missed in working samples, compromising the *Amphimerus* sp. DNA obtained and thus subsequent amplification. It is also important to note that we established the minimum amount of *Amphimerus* sp. genomic DNA detectable by LAMP is 0.001 ng (1 pg). It has been reported that a single egg of a closely related trematode *O*. *viverrini* yields 3.72 ng of genomic DNA [45]. Then, theoretically, our LAMP assay would detect *Amphimerus* sp. DNA corresponding to less than one single egg in a stool sample. Another possibility could have been a mistake in the morphological identification of parasite eggs when performing the stool microscopic examination. This observation would further confirm the specificity of LAMPhimerus method in the amplification of *Amphimerus* sp. DNA alone.

However, as noted elsewhere, the need for a decision in case management dictates unequivocal result interpretation [22] and some of the drawbacks of LAMP assays, such as potential DNA contamination and carry-over of amplified products when opening the tubes to use the dye, should be considered because they may compromise the test results.

In summary, we have developed, for the first time, a LAMP assay (namely, LAMPhimerus) for the sensitive and specific detection of *Amphimerus* sp. DNA in human stool samples. After further research for validation, the method could be readily adapted for effective field diagnosis and disease surveillance in amphimeriasis-endemic areas. Future work will be aimed at large-scale studies to further assess the applicability of this novel diagnostic tool.

## Supporting information

S1 Fig Examination of human stool samples by PCR F3-B3. Analysis of human stool samples included in the study by PCR using outer primers F3 and B3 to detect *Amphimerus* sp. DNA. In all panels: lane Amp, DNA of *Amphimerus* sp. (10 ng); lane M, molecular weight marker (100 bp Plus Blue DNA Ladder); lane N, negative control (ultrapure water, no DNA template); and numbers 5–104, stool samples analyzed.
(TIF)

## References

[1] Yamaguti S. Synopsis of the digenetic trematodes of vertebrates. Vols. 1 and II Tokyo: Keigaku Co; 1971 p. 1074.

[2] Bowman DD. *Amphimerus pseudofelineus* (Ward 1901) Barker, 1911 In: Feline clinical parasitology. 1st ed Ames (IA): Iowa State University Press 2002; p. 151–153.

[3] Calvopiña M, Cevallos W, Kumazawa H, Eisenberg J. High prevalence of human liver infection by *Amphimerus* spp. Flukes, Ecuador. Emerg Infect Dis. 2011; 17: 2331–2334. doi: 10.3201/eid1712.110373 22172165 10.3201/eid1712.110373 PMC3311191

[4] Calvopiña M, Cevallos W, Atherton R, Saunders M, Small A, Kumazawa H, et al High prevalence of the liver fluke *Amphimerus* spp. In domestic cats and dogs in an area for human amphimeriasis in Ecuador. PLos Negl Trop Dis. 2015; 9: e0003526 doi: 10.1371/journal.pntd.0003526 25647171 10.1371/journal.pntd.0003526 PMC4315407

[5] World Health Organization. 2015. “Investing to overcome the global impact of neglected tropical diseases—Third WHO report on neglected tropical diseases”. http://www.who.int/neglected_diseases/9789241564861/en/

[6] Rim HJ. Clonorchiasis: an update. J. Helminthol. 2005; 79: 269–281. 16153321 10.1079/joh2005300

[7] Sripa B, Kaewkes S., Sithithaworn P, Mairiang E, Laha T, Smout M et al Liver fluke induces colangiocarcinoma. PLos Negl Trop Dis. 2007; 4: 1148–55. 10.1371/journal.pmed.0040201 PMC1913093 17622191

[8] Bouvard V, Baan R, Straif K, Grosse Y, Secretan B, EI Ghissassi F, et al A review of human carcinogens-Part B: biological agents. Lancet. Oncol. 2009; 10: 321–322. 19350698 10.1016/s1470-2045(09)70096-8

[9] Parvathi A, Kumer HA, Prakasha BK, Lu J, Xu X, Hu W, et al *Clonorchis sinensis*: development and evaluation of a nested polymerase chain reaction (PCR) assay. Exp Parasitol. 2007; 115: 291–295. doi: 10.1016/j.exppara.2006.09.010 17067580 10.1016/j.exppara.2006.09.010

[10] Kim EM, Verweij JJ, Jalili A, van Lieshout L, Choi MH, Bae YM, et al Detection of *Clonorchis sinensis* in stool samples using real-time PCR. Ann Trop Med Parasitol. 2009; 103: 513–518. doi: 10.1179/136485909X451834 19695156 10.1179/136485909X451834

[11] Rahman SM, Bae YM, Hong ST, Choi MH. Early detection and estimation of infection burden by real-time PCR in rats experimentally infected with *Clonorchis sinensis*. Parasitol Res. 2011; 109: 297–303. doi: 10.1007/s00436-011-2253-3 21279385 10.1007/s00436-011-2253-3

[12] Cai XQ, Yu HQ, Bai JS, Tang JD, Hu XC, Chen DH, et al Development of a TaqMan based real-time PCR assay for detection of *Clonorchis sinensis* DNA in human stool samples and fishes. Parasitol Int. 2012; 61: 183–186. doi: 10.1016/j.parint.2011.06.010 21729765 10.1016/j.parint.2011.06.010

[13] Sanpool O, Intapan PM, Thanchomnang T, Janwan P, Lulitanond V, Doanh PN, et al Rapid detection and differentiation of *Clonorchis sinensis* and *Opisthorchis viverrini* eggs in human fecal samples using a duplex real-time fluorescence resonance energy transfer PCR and melting curve analysis. Parasitol Res. 2012; 111: 89–96. doi: 10.1007/s00436-011-2804-7 22246366 10.1007/s00436-011-2804-7

[14] Huang SY, Tang JD, Song HQ, Fu BQ, Xu MJ, Hu XC, et al A specific PCR assay for the diagnosis of *Clonorchis sinensis* infection in humans, cats and fishes. Parasitol Int. 2012; 61: 187–190. doi: 10.1016/j.parint.2011.07.010 21777693 10.1016/j.parint.2011.07.010

[15] Wongratanacheewin S, Pumidonming W, Sermswan RW, Pipitgool V, Maleewong W. Detection of *Opisthorchis viverrini* in human stool specimens by PCR. J Clin Microbiol. 2002; 40: 3879–3880. doi: 10.1128/JCM.40.10.3879-3880.2002 12354909 10.1128/JCM.40.10.3879-3880.2002 PMC130907

[16] Muller B, Schmidt J, Mehlhorn H. PCR diagnosis of infections with different species of Opisthorchiidae using a rapid clean-up procedure for stool samples and specific primers. Parasitol Res. 2007; 100: 905–909. doi: 10.1007/s00436-006-0321-x 17061114 10.1007/s00436-006-0321-x

[17] Lovis L, Mak TK, Phongluxa K, et al PCR Diagnosis of *Opisthorchis viverrini* and *Haplorchis taichui* Infections in a Lao Community in an Area of Endemicity and Comparison of Diagnostic Methods for Parasitological Field Surveys. J Clin Microbiol. 2009; 47: 1517–1523. doi: 10.1128/JCM.02011-08 19279176 10.1128/JCM.02011-08 PMC2681877

[18] Kaewkong W, Intapan PM, Sanpool O, et al Molecular Differentiation of *Opisthorchis viverrini* and *Clonorchis sinensis* Eggs by Multiplex Real-Time PCR with High Resolution Melting Analysis. Korean J Parasitol. 2013; 51: 689–694. doi: 10.3347/kjp.2013.51.6.689 24516275 10.3347/kjp.2013.51.6.689 PMC3916459

[19] Notomi T, Okayama H, Masubuchi H, Yonekawa T, Watanabe K, Amino N, et al Loop-mediated isothermal amplification of DNA. Nucleic Acids Res. 2000; 28: E63 10871386 10.1093/nar/28.12.e63 PMC102748

[20] Zhang X, Lowe SB, Gooding JJ. Brief review of monitoring methods for loop-mediated isothermal amplification (LAMP). Biosens Bioelectron. 2014; 61: 491–499. doi: 10.1016/j.bios.2014.05.039 24949822 10.1016/j.bios.2014.05.039

[21] Notomi T, Mori Y, Tomita N, Kanda H. Loop-mediated isothermal amplification (LAMP): principle, features, and future prospects. J Microbiol. 2015; 53: 1–5. doi: 10.1007/s12275-015-4656-9 25557475 10.1007/s12275-015-4656-9

[22] Njiru ZK. Loop-mediated isothermal amplification technology: towards point of care diagnostics. PLoS Negl Trop Dis. 2010; 6: e1572. 10.1371/journal.pntd.0001572 PMC3383729 22745836

[23] Mori Y, Kanda H, Notomi T. Loop-mediated isothermal amplification (LAMP): recent progress in research and development. J Infect Chemother. 2013; 19: 404–411. 23539453 10.1007/s10156-013-0590-0 PMC7088141

[24] Ai L, Li C, Elsheikha HM, Hong SJ, Chen JX, Chen SH, Li X, Cai XQ, Chen MX, Zhu XQ. Rapid identification and differentiation of *Fasciola hepatica* and *Fasciola gigantica* by a loop-mediated isothermal amplification (LAMP) assay. Vet Parasitol. 2010; 174: 228–233. doi: 10.1016/j.vetpar.2010.09.005 20933335 10.1016/j.vetpar.2010.09.005

[25] Cai XQ, Xu MJ, Wang YH, Qiu DY, Liu GX, Lin A, Tang JD, Zhang RL, Zhu XQ. Sensitive and rapid detection of *Clonorchis sinensis* infection in fish by loop-mediated isothermal amplification (LAMP). Parasitol Res. 2010; 106: 1379–1383. doi: 10.1007/s00436-010-1812-3 20232082 10.1007/s00436-010-1812-3

[26] Chen Y, Wen T, Lai DH, Wen YZ, Wu ZD, Yang TB, Yu XB, Hide G, Lun ZR. Development and evaluation of loop-mediated isothermal amplification (LAMP) for rapid detection of *Clonorchis sinensi*s from its first intermediate hosts, freshwater snails. Parasitology. 2013; 140: 1377–1383. doi: 10.1017/S0031182013000498 23870065 10.1017/S0031182013000498

[27] Arimatsu Y, Kaewkes S, Laha T, Hong SJ, Sripa B. Rapid detection of *Opisthorchis viverrini* copro-DNA using loop-mediated isothermal amplification (LAMP). Parasitol Int. 2012; 61: 178–182. doi: 10.1016/j.parint.2011.08.009 21871581 10.1016/j.parint.2011.08.009 PMC4444511

[28] Le TH, Nguyen NT, Truong NH, De NV. Development of mitochondrial loop-mediated isothermal amplification for detection of the small liver fluke *Opisthorchis viverrini* (Opisthorchiidae; Trematoda; Platyhelminthes). J Clin Microbiol. 2012; 50: 1178–1184. doi: 10.1128/JCM.06277-11 22322346 10.1128/JCM.06277-11 PMC3318558

[29] Arimatsu Y, Kaewkes S, Laha T, Sripa B. Specific diagnosis of *Opisthorchis viverrini* using loop-mediated isothermal amplification (LAMP) targeting parasite microsatellites. Acta Trop. 2015; 141: 368–371. doi: 10.1016/j.actatropica.2014.09.012 25268466 10.1016/j.actatropica.2014.09.012 PMC4454772

[30] Chen MX, Ai L, Zhang RL, Xia JJ, Wang K, Chen SH, et al Sensitive and rapid detection of *Paragonimus westermani* infection in humans and animals by loop-mediated isothermal amplification (LAMP). Parasitol Res. 2011; 108: 1193–1198. doi: 10.1007/s00436-010-2162-x 21107864 10.1007/s00436-010-2162-x

[31] Sierra R. Traditional resource-use systems and tropical deforestation in a multi-ethnic region in North-west Ecuador. Environ Conserv. 1999; 26: 136–145.

[32] Myers N, Mittermeier RA, Mittermeier CG, da Fonseca GAB, Kent J. Biodiversity hotspots for conservation priorities. Nature. 2000; 403: 853–8. doi: 10.1038/35002501 10706275 10.1038/35002501

[33] World Health Organization. Basic laboratory methods in medical parasitology. World Health Organization Publications, Geneva, Switzerland, 1992 ISBN 92-4-15410-4. 114 pp.

[34] Altschul SF, Gish W, Miller W, Myers EW, Lipman DJ. Basic local alignment search tool. J Mol Biol. 1990; 215: 403–410. doi: 10.1016/S0022-2836(05)80360-2 2231712 10.1016/S0022-2836(05)80360-2

[35] Johansen MV, Sithithaworn P, Bergquist R, Utzinger J. Towards improved diagnosis of zoonotic trematode infections in Southeast Asia. Adv Parasitol. 2010; 73: 171–195 doi: 10.1016/S0065-308X(10)73007-4 20627143 10.1016/S0065-308X(10)73007-4

[36] Esteban JG, Muñoz-Antoli C, Toledo R, Ash LR. Diagnosis of human trematode infections. Adv Exp Med Biol. 2014; 766: 293–327. doi: 10.1007/978-1-4939-0915-5_9 24903369 10.1007/978-1-4939-0915-5_9

[37] Johansen MV, Lier T, Sithithaworn P. Towards improved diagnosis of neglected zoonotic trematodes using a One Health approach. Acta Trop. 2015;141: 161–169. doi: 10.1016/j.actatropica.2013.07.006 23886849 10.1016/j.actatropica.2013.07.006

[38] Parida M.M, Horioke K, Ishida H, Dash P.K, Saxena P, Jana A.M, Islam M.A, Inoue S, Hosaka N, Morita K. Rapid detection and differentiation of dengue virus serotypes by a real-time reverse transcription-loop-mediated isothermal amplification assay. J. Clin. Microbio. 2005; 43: 2895–2903. 10.1128/JCM.43.6.2895-2903.2005 PMC1151941 15956414

[39] Abu Al-Soud W., and Radstrom P.. Effects of amplification facilitators on diagnostic PCR in the presence of blood, feces, and meat. J. Clin. Microbiol. 2000; 38: 4463–4470. 11101581 10.1128/jcm.38.12.4463-4470.2000 PMC87622

[40] Duenngai K, Sithithaworn P, Rudrappa UK, Iddya K, Laha T, Stensvold CR, Strandgaard H, Johansen MV. Improvement of PCR for detection of *Opisthorchis viverrini* DNA in human stool samples. J Clin Microbiol. 2008; 46: 366–368. doi: 10.1128/JCM.01323-07 18003810 10.1128/JCM.01323-07 PMC2224308

[41] Kaneko H, Kawana T, Fukushima E, Suzutani T. Tolerance of loop-mediated isothermal amplification to a culture medium and biological substances. J Biochem Biophys Methods. 2007; 70: 499–501. doi: 10.1016/j.jbbm.2006.08.008 17011631 10.1016/j.jbbm.2006.08.008

[42] Francois P, Tangomo M, Hibbs J, Bonetti EJ, Boehme CC, Notomi T, et al Robustness of loop-mediated isothermal amplification reaction for diagnostics applications. FEMS Immunol Med Microbiol. 2011; 62: 41–48. doi: 10.1111/j.1574-695X.2011.00785.x 21276085 10.1111/j.1574-695X.2011.00785.x

[43] Geojith G, Dhanasekaran S, Chandran S, Kenneth J. Efficacy of loop mediated isothermal amplification (LAMP) assay for the laboratory identification of *Mycobacterium tuberculosis* isolates in a resource limited setting. J Microbiol Methods. 2011; 84: 71–73. doi: 10.1016/j.mimet.2010.10.015 21047534 10.1016/j.mimet.2010.10.015

[44] Levecke B, Behnke JM, Ajjampur SS, Albonico M, Ame SM, Charlier J, et al A comparison of the sensitivity and fecal egg counts of the McMaster egg counting and Kato-Katz thick smear methods for soil-transmitted helminths. PLoS Negl Trop Dis. 2011; 5: e1201 doi: 10.1371/journal.pntd.0001201 21695104 10.1371/journal.pntd.0001201 PMC3114752

[45] Wongsaward Ch, Wongsaward P, Chai J-Y, Paratasilpin T, Anuntalabhochai S. DNA quantities and qualities from various stages of some trematodes using opical and HAT-RAPD methods. Southeast Asian J Trop Med Public Health. 2006; 37: 62–68. 17547055

