# Supplementary figures and images for: LAMPhimerus: A novel LAMP assay for detecting Amphimerus sp. DNA in human stool samples
Source: PLoS Negl Trop Dis. 2017 Jun 19;11(6):e0005672. doi: 10.1371/journal.pntd.0005672 (PMC5491318; doi:10.1371/journal.pntd.0005672)

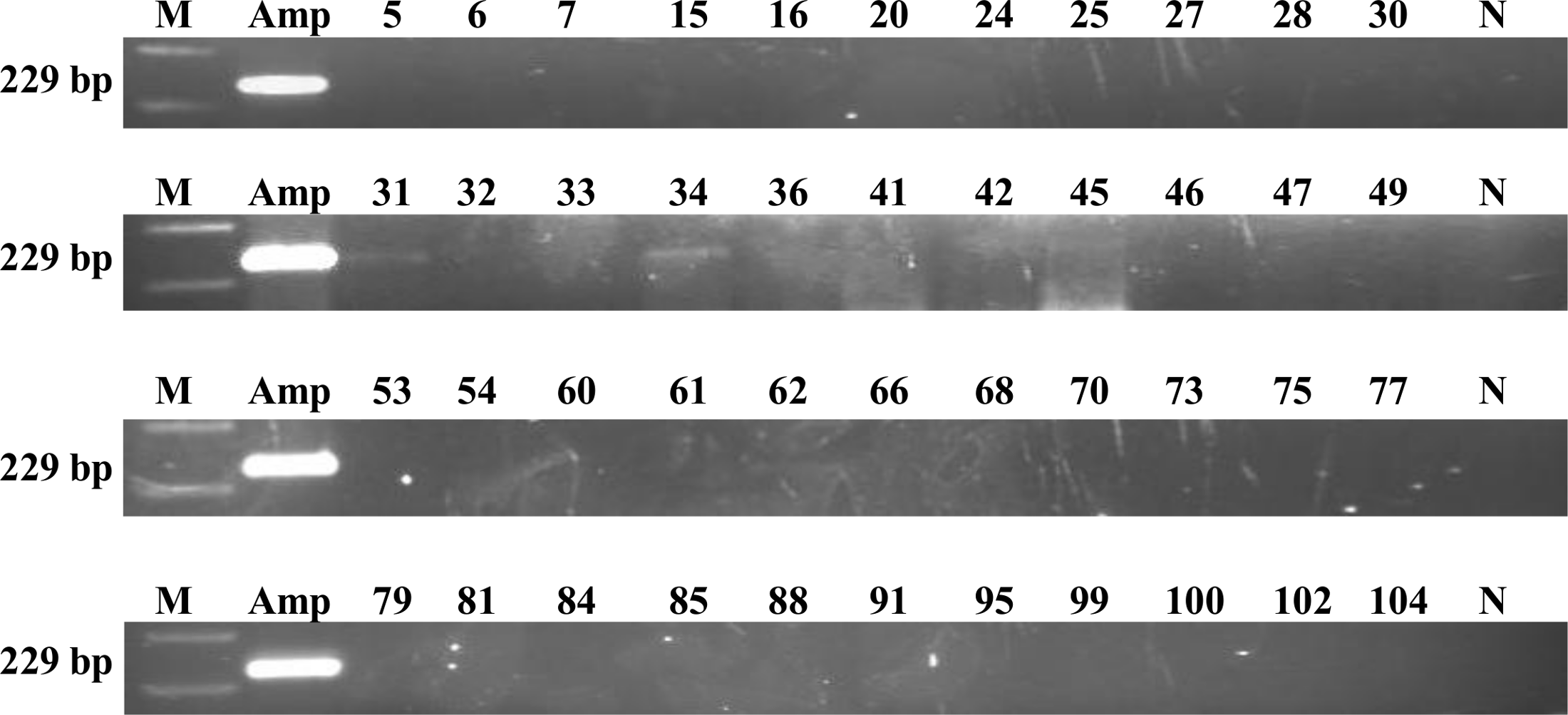

Supplement: S1 Fig — Analysis of human stool samples included in the study by PCR using outer primers F3 and B3 to detect Amphimerus sp. DNA. In all panels: lane Amp, DNA of Amphimerus sp. (10 ng); lane M, molecular weight marker (100 bp Plus Blue DNA Ladder); lane N, negative control (ultrapure water, no DNA template); and numbers 5–104, stool samples analyzed. (TIF) [file pntd.0005672.s001.tif]
